# Innovative use of indigenous *dadih* probiotics to enhance feed intake, digestibility, growth performance, and health in heat-stressed Sapera goats

**DOI:** 10.14202/vetworld.2025.1224-1233

**Published:** 2025-05-17

**Authors:** Nurzainah Ginting, Edhy Mirwandhono, Nurjama’yah Br. Ketaren, Yuan-Yu Lin

**Affiliations:** 1 Study Program of Animal Science, Faculty of Agriculture, Universitas Sumatera Utara, Medan 20155, Indonesia; 2 Department of Animal Science and Technology, National Taiwan University, Taipei, Taiwan

**Keywords:** *dadih*, feed efficiency, growth performance, gut microbiota, heat stress, indigenous probiotic, *Lactiplantibacillus plantarum*, Sapera goats

## Abstract

**Background and Aim:**

Heat stress resulting from rising ambient temperatures in tropical climates poses a significant threat to ruminant productivity, leading to suppressed feed intake, impaired growth, and reduced health. Indigenous fermented foods such as *dadih* – a traditional probiotic made from fermented buffalo milk in bamboo tubes – may offer a sustainable nutritional intervention. This study aimed to investigate the effects of *dadih* supplementation on feed consumption, nutrient digestibility, growth performance, pathogenic bacterial load, and hematological profiles of heat-stressed Sapera goats.

**Materials and Methods:**

The dominant bacterial strain in *dadih* was characterized using 16S ribosomal RNA sequencing and evaluated for *in vitro* antagonism against *Escherichia coli* and *Salmonella* spp. An *in vivo* trial was conducted using 15 Sapera crossbred goats (15 ± 1.46 kg), randomly allocated into three treatment groups (n = 5): 8 cc *dadih*/day (Group A), 4 cc/day (Group B), and control (Group C). The trial lasted 4 weeks, during which feed consumption, daily weight gain, feed efficiency (FE), fecal pathogenic bacteria load, and hematological parameters were measured.

**Results:**

The probiotic strain was identified as *Lactiplantibacillus plantarum* Japan collection of microorganisms 1149, exhibiting antibacterial activity with inhibition zones of 9.3 mm (*E. coli*) and 9.5 mm (*Salmonella*). Goats supplemented with 4 cc *dadih* (Group B) demonstrated the highest daily weight gain (127.14 g/day), FE (0.15), and nutrient digestibility. A higher *dadih* dose (8 cc) significantly reduced fecal *E. coli* levels. Hematological indices remained within normal physiological ranges across all treatments, suggesting no adverse effects.

**Conclusion:**

This study provides the first empirical evidence supporting the use of *dadih* as a climate-adaptive probiotic intervention in goats. Supplementation with 4 cc *dadih* optimized performance without disrupting hematological homeostasis, while 8 cc effectively suppressed gut pathogens. These findings offer novel insights into the functional role of traditional fermented probiotics in improving resilience to heat stress and promoting sustainable small ruminant production in tropical environments.

## INTRODUCTION

Dairy goat farming represents a vital agricultural sector in Indonesia, largely due to the adaptability of goats to tropical climates. The Sapera goat is a cross-breed developed from the Etawa Crossbreed (itself a hybrid of Jamnapari and Kacang goats) and the Saanen goat, selectively bred for high milk yield and efficient feed conversion [1, 2]. Although Sapera goats are relatively smaller in stature, they outperform Etawa crossbreeds in milk production while requiring less feed [3]. Farmers generally aspire for optimal growth in goats from an early stage, which is achievable through the provision of high-quality feed. However, despite good nutritional support, climate change – particularly elevated ambient temperatures – poses significant challenges by inducing heat stress [4, 5].

**Figure 1:**
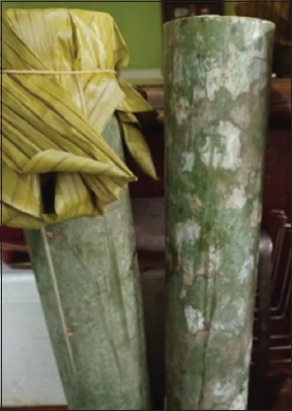
*Dadih* is fermented in bamboo tubes.

To mitigate the effects of heat stress in goats, the use of probiotics is recommended by Cai *et al*. [6], with *dadih* serving as a promising candidate. *Dadih* is a highly nutritious traditional food that is easily absorbed by the body, containing 16 amino acids (13 essential and three non-essential). It is also rich in vitamins, including vitamin A (1.70–7.22 IU/g), along with vitamins B and K [7]. In general, *dadih* contains high levels of both protein and fat, with an average protein content of 6.75% [8]. It is traditionally produced by fermenting buffalo milk in bamboo tubes (Figure 1), a practice rooted in Indonesian local wisdom that dates back centuries.

**Figure 2:**
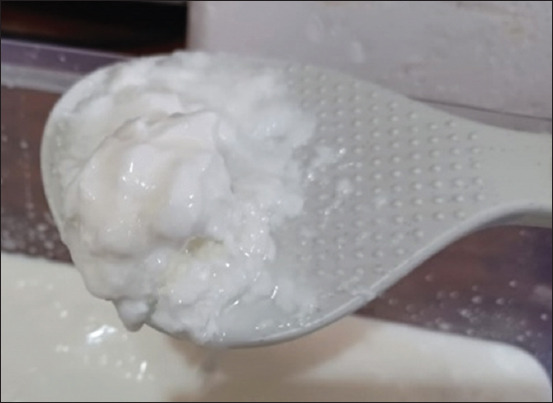
The *dadih* clots have passed the fermentation process.

Lactic acid bacteria (LAB), known for their health benefits, are prevalent in *dadih*. Fermentation is facilitated by microorganisms naturally present in milk [9] and typically lasts for 24 h, yielding a white, lumpy product (Figure 2). In addition to milk, bamboo segments also contribute microbial flora; the dominant genera identified include *Lactobacillus*, *Leuconostoc*, *Lactococcus*, *Streptococcus*, and *Enterococcus* [10]. Fermented milk is considered one of the classical sour-ces of probiotics, offering a range of clinical benefits for young livestock, and has been widely adopted for such applications [11].

Despite the increasing interest in utilizing probi-otics to mitigate heat stress and improve ruminant productivity, most probiotic applications in livestock have focused on commercially available microbial strains, with limited attention given to indigenous fermented products. *Dadih*, a traditional Indonesian fermented milk, harbors a diverse microbial population, including LAB with established health-promoting properties. However, its application in small ruminants, particularly under climate-induced stress conditions, remains largely underexplored. Moreover, there is a paucity of *in vivo* studies that quantify the physiological and microbiological responses of goats to *dadih* supplementation, especially under high ambient tempe-rature regimens. The potential of *dadih* to serve as a locally sourced, low-cost functional feed additive for improving feed utilization, suppressing gut pathogens, and maintaining systemic health in heat-stressed goats has not been rigorously investigated in a controlled setting.

This study aimed to evaluate the effects of *dadih* supplementation on feed intake, growth performance, feed efficiency (FE), fecal pathogenic bacteria load, and hematological parameters in Sapera goats exposed to heat stress. Specifically, the research sought to characterize the dominant probiotic strains present in *dadih*, assess their *in vitro* antibacterial activity, and determine the *in vivo* efficacy of varying *dadih* doses in enhancing productivity and health outcomes under extreme temperature conditions. By integ-rating traditional knowledge with scientific evaluation, this study aspires to promote the use of indigenous probiotics as a sustainable solution for improving resilience and performance in tropical goat farming systems.

## MATERIALS AND METHODS

### Ethical approval

All experimental procedures were approved and monitored by the Research Ethics Committee of the Faculty of Mathematics and Natural Sciences, Universitas Sumatera Utara (approval number: 0478/KEPH-FMIPA/2024).

### Study period and location

The study was conducted at the Roemah Susu Etawa Abizar Dairy Goat Farm, located in Deli Serdang Regency, North Sumatra Province, Indonesia (https://maps.app.goo.gl/YjBbGV5cJjtPx7Rf6). The experimental period lasted from January to June 2024. Laboratory analyses were conducted at the Laboratory of Animal Production and Microbiology, Faculty of Agriculture, Universitas Sumatera Utara. The 16S ribosomal RNA (16S rRNA) sequencing was performed by Macrogen Singapore, and blood samples were analyzed at the Veterinary Center, Bukittinggi, West Sumatra, Indonesia.

### Experimental procedure and probiotic preparation

This study represents the first attempt to isolate the dominant bacterial strain from *dadih*. The isolated bacteria were sequenced and submitted to GenBank. Antibacterial activity was assessed *in vitro* against *Escherichia coli* and *Salmonella* spp., common gut pathogens in goats that, if unregulated, can induce diarrhea.

### Experimental design and animals

A total of fifteen growing Sapera crossbred goats (15 ± 1.46 kg) were selected and randomly assigned into three treatment groups (n = 5 per group) using a randomized block design with five replications. Before the trial, all goats underwent deworming and a 14-day adaptation period. The experimental treatments were as follows:

- Group A: *Dadih* 8 cc/head/day
- Group B: *Dadih* 4 cc/head/day
- Group C: Control (0 cc/head/day).

Each goat was individually housed and provided with *ad libitum* access to clean drinking water.

### Feeding and *dadih* administration

**Figure 3:**
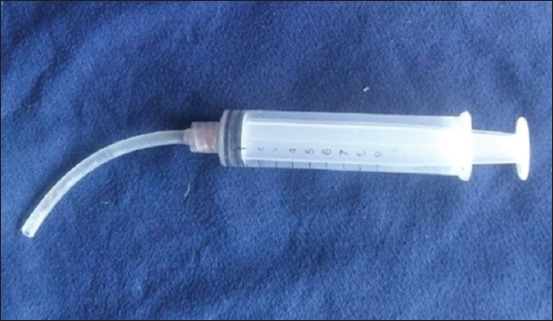
A 10-cc syringe with a small infusion hose was used to feed the *dadih* to the goats.

*Dadih* was administered orally at 07:45 AM using a custom syringe-based device (Figure 3). The *dadih* was drawn into a 10-cc syringe fitted with a 10-cm infusion hose in place of a needle. The tube was inserted through the lateral jawline into the posterior oral cavity to ensure accurate delivery. The hose length was tailored to the jaw dimensions of 5-month-old Sapera goats.

Following *dadih* administration, concentrate feed (3% of body weight on a dry matter basis) was provided in two equal portions at 08:00 and 16:00. Before the experimental period, the concentrate was supplied at 2.5% but was increased to 3% based on farmer feedback to avoid economic constraint post-study. Forage was provided as chopped yam leaves (3–5 cm) [12], which were sun-dried and fermented overnight in sealed plastic barrels to reduce cyanide levels [13, 14]. Forage was offered at 4% of body weight (dry matter) in two daily feedings following concentrate provision.

### Performance monitoring and sample collection

Feed refusals were measured daily to assess feed intake, while body weights were recorded every 3 days to monitor changes under fluctuating temperatures. A notable weight decline was observed in goats not supplemented with *dadih* during peak ambient temperatures (37°C).

At the conclusion of the study, blood samples were collected at 08:00 AM from the jugular vein using sterile equipment (3 mL syringe, ethylenediaminetetraacetic acid vacuum tube, 70% alcohol, and sterile cotton). Samples were mixed gently, labeled, and stored in a cool box before hematological analysis [15].

Fecal samples were collected rectally using gentle digital palpation. Samples were placed in sterile plastic containers, labeled, stored in a fecal cool box, and immediately transported to the laboratory for quantification of *E. coli* and *Salmonella* populations.

### Experimental parameters

Measured parameters included:

- Feed consumption
- Daily body weight gain (DBWG)
- FE
- Fecal pathogenic bacteria population
- Hematological profile.

The composition of the experimental diets and feed chemical analysis are presented in Tables 1 and 2.

**Table 1:** Concentrated feed ingredients.

Feed ingredients	Proportion (%)
Composition of feed ingredients	
Palm kernel cake	15.00
Soybean groats	27.00
Soybean hulls	15.00
Rice bran	8.90
Corn hulls	13.00
Potato dregs	12.00
Coffee hulls	8.00
Salt	0.20
Toxin binder	0.10
Vitamin complex	0.20
Premix	0.10
Feed flavor	0.10
Rock flavor	0.30
MCP	0.10
Total	100
Concentrate nutrient content (% DM)	
DM	100
Crude protein	15.52
Crude fiber	14.47
Crude fat	4.33
Ash	8.41
BETN	51.24
TDN	66.54

TDN=Total digestible nutrient, BETN=Basic exchangeable nutrient table, DM=Dry matter, MCP=Mono Calcium Phosphate

**Table 2:** Description of *Lactiplantibacillus plantarum* JCM 1149

Sequencing Primer name Primer sequences					PCR Primer name Primer sequences				
785F 5’ (GGA TTA GAT ACC CTG GTA) 3’					27F 5’ (AGA GTT TGA TCM TGG CTC AG) 3’				
907R 5’ (CCG TCA ATT CMT TTR AGT TT) 3’					1492R 5’ (TAC GGY TAC CTT GTT ACG ACT T) 3’				

**Subject**						**Score**		**Identities**	
		
**Accession**	**Description**	**Length**	**Start**	**End**	**Coverage**	**Bit**	**E-Value**	**Match/Total**	**Pct.(%)**

NR_ 115695.1	*Lactiplantibacillus plantarum*	1519	15	1490	97	2726	0.0	1476/1476	100.00

JCM=Japan collection of microorganisms

### Statistical analysis

All data were analyzed using analysis of variance followed by Duncan’s multiple range test to determine significant differences between treatment groups (p < 0.05), using the Statistical Package for the Social Sciences version 27.0 (IBM Corp., NY, USA).

## RESULTS

### Isolation and characterization of LAB from *dadih*

#### Total LAB and molecular identification

The total LAB population in *dadih* was deter-mined to be 1.12 × 10^10^ colony-forming unit (CFU)/mL. Several Gram-positive LAB strains were isolated and subsequently sent to Macrogen (Singapore) for mole-cular identification using *16S rRNA* gene polymerase chain reaction analysis. The results revealed that the dominant genus was *Lactiplantibacillus*, specifically identified as *Lactiplantibacillus* *plantarum* strain Japan collection of microorganisms (JCM) 1149, which was registered in the GenBank database of the National Center for Biotechnology Information (NCBI) (Table 2). According to the NCBI taxonomic update as of October 2022, the genus *Lactobacillus* has been reclassified into 22 genera, one of which is *Lactiplantibacillus*. The identified species, *L. plantarum* JCM 1149, is recognized as a probiotic.

#### Antibacterial assay of LAB isolates

The antibacterial activity of *L. plantarum* JCM 1149 was evaluated *in vitro* against *E. coli* and *Salmonella* spp. isolated from goat housing environments. The inhibition zones observed were 9.3 mm for *E. coli* and 9.5 mm for *Salmonella*, indicating the antibacterial potential of the isolated probiotic strain.

### Feed intake and dietary preferences

#### Dadih feeding and behavioral response

*Dadih* supplementation was administered once daily before 08:00 AM. Goats that received *dadih* displayed a noticeable preference for it, often waiting in anticipation as the handler approached. The *dadih* was delivered using a syringe fitted with a flexible hose, inserted through the lateral edge of the mouth to the posterior oral cavity, past the last molar.

#### Concentrate and forage consumption

Concentrate feed was provided at 3% of body weight on a dry matter basis, divided into two equal feedings at 08:00 and 16:00. The feed was consistently consumed in full across treatments.

The forage provided consisted of chopped yam leaves. The quantity of forage consumed is presented in Table 3. Statistical analysis indicated that forage intake in Groups A (8 cc *dadih*) and B (4 cc *dadih*) was significantly higher (p < 0.05) compared to the control group (Group C).

**Table 3:** Ration nutrients consumption and digestibility during extreme temperatures.

Items	Treatments		

	A	B	C
DMI (g/head/day)	825.973^a^ ± 5.02	834.37^a^ ± 3.02	829.24^a^ ± 2.03
CPI (g/head/day)	97.35^b^ ± 1.1	103.50^c^ ± 2.25	86.67^a^ ± 3.7
Dry matter digestibility (%)	73.11^b^ ± 1.1	75.76^b^ ± 1.65	67.78^a^ ± 1.27
Digestibility of organic matter (%)	73.15^b^ ± 0.85	75.17^b^ ± 1.03	65.01^a^ ± 2.99
TDN (%)	74.16^b^ ± 3.04	77.00^b^ ± 0.88	67.22^a^ ± 3.07

Notes: The same superscript in the same column indicates not significantly different (p > 0.05), DMI=Dry matter intake, CPI=Crude protein intake, TDN=Total digestible nutrient

### Assessment of pathogenic bacteria

#### In vitro and in vivo evaluation

The *in vitro* antibacterial test confirmed that *dadih* effectively inhibited the growth of pathogenic *E. coli* and *Salmonella* through the presence of measurable inhibition zones. To assess the *in vivo* effect, fecal samples from experimental goats were examined for pathogenic bacterial populations.

Fecal collection involved gentle rectal scraping, after which the samples were immediately placed in sterile plastic containers, stored in a cool box, and transported to the laboratory for analysis of *E. coli* and *Salmonella* populations.

## DISCUSSION

### Total LAB in *dadih* and antibacterial test

The total number of LAB in this study is 1.12 × 10^10^ CFU/mL and is dominated by the genus *Lactobacillus*. A study by Ary *et al*. [16] showed that the LAB population was 3.63 × 10^8^ CFU/mL, while the dominant genus was Lactococcus. Many factors influence the dominant genus, especially location, such as the results of research by Yu *et al*. [17] and Gao *et al*. [18] in Mongolia dominated by *Lactobacillus*, Zhang *et al*. [19] in China by *Lactobacillus*, Jans *et al*. [20] in Africa by *Streptococcus*, Mechai *et al*. [21] in Algeria by *Lactobacillus*, Abed [22] and Yu *et al*. [23] in Russia by *Lactobacillus*, Yu *et al*. [24], Liu *et al*. [25] and Ren and Suo [26] in Tibet by *Lactobacillus*, Shangpliang *et al*. [27] by Lactococcus and *Lactobacillus* in India, and Ehsani *et al*. [28] in the dominant genus by *Lactobacillus*. It can be concluded that in dairy products where spontaneous fermentation occurs, the genus of LAB found is predominantly *Lactobacillus*. Zendeboodi *et al*. [29] also stated that the microbes most widely used in food fermentation processes are LAB, especially the *Lactobacillus* and *Bifidobacteriaceae* genera.

At present, *Lactobacillus* consists of 200 species that play a role in the fermentation of foods. Exploration of this genus must continue, considering its health benefits for humans and animals. Many of its strains are declared probiotics, indicating they exhibit health benefits beyond basic nutritional value [30].

In this study, antibacterial tests were conducted by testing the supernatant of *Lactobacillus* *plantarum* JCM 1149 against pathogenic bacteria isolated from goat sheds, namely *E. coli* and *Salmonella*. The results showed that the antibacterial inhibition zone against *E. coli* was 9.3 mm, while against *Salmonella*, it was 9.5 mm. Research conducted by Girma and Aemiro [31] found that the antibacterial zone of *L. plantarum* isolated from dairy products in Ethiopia against *E. coli* was not detected. In contrast, Gao *et al*. [18] found inhibition zones of approximately 11.0 mm for *E. coli* and 12.0 mm for *Salmonella* from *dadih* isolated from buffalo milk. According to Shafique *et al*. [32], *L. plantarum* as an LAB produces antimicrobial agents such as bacteriocins, diacetyl, organic acids, carbon dioxide, and hydrogen peroxide. These agents play a role in inhibiting the growth of pathogenic bacteria, enabling LAB to maintain livestock health.

### Feed consumption

Climate change has been recognized as a harmful factor affecting goat production. Small ruminants are vulnerable to the direct and indirect impacts of climate change, including heat stress. Extreme temperature significantly influences consumption levels. Feed consumption usually decreases with increasing tempe-rature. The higher the ambient temperature, the more overheating occurs in the body of livestock, resulting in reduced feed demand.

However, consumption is related to digestibility, which is influenced by the microbial atmosphere of the rumen [33]. In this study, the highest consumption was found in treatment B, i.e., 4 cc *dadih*, compared with other treatments – 8 cc and without *dadih* (Table 3). The digestibility of dry and organic matter was highest following treatment with 4 cc *dadih* (Table 3). *Dadih* is a probiotic that contains beneficial bacteria, and the population of LAB in this study was 1.12 × 10^10^ CFU/mL. In *dadih*, there are cellulase-producing bacteria, likely originating from the bamboo container, which cont-ains cellulose. The presence of milk in bamboo tubes supports the growth of these bacteria, which in turn produce cellulase enzymes.

**Figure 4:**
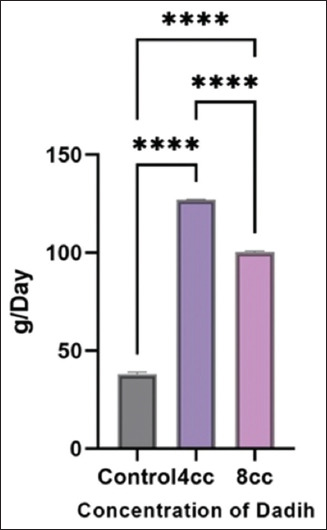
Effect of dadih feeding on the daily body weight gain of Sapera goats at extreme temperatures.

Treatment with 4 cc *dadih* increased feed diges-tibility by helping to break down cellulose (Table 3). The presence of proteolytic bacteria helped prot-ein degradation, thereby increasing nitrogen intake. The microbial population in *dadih*, dominated by LAB, produced lactic acid, creating a more acidic atmosphere in the rumen. This acidic environment inhibited the development of pathogenic bacteria in the rumen, establishing balance in the rumen ecosystem. As a result, nutrient absorption improved, contributing to increased average daily gain (ADG) (Figure 4) and FE (Table 4). Giving 8 cc *dadih* made the ruminal environment more acidic, which inhibited not only pathogenic but also non-pathogenic bacteria. In the control treatment, although consumption was good, the ration digestibility was the lowest due to limited cellulose breakdown.

**Table 4:** Daily body weight gain and feed utilization efficiency in Sapera goats at extreme temperatures.

Treatments	Variables			

	IBW (kg)	FBW (kg)	DBWG (g)	FE (%)
A	17.44^a^ ± 1.73	19.53^b^ ± 2.73	100.00^b^ ± 0.62	0.12^b^ ± 0.1
B	14.71^a^ ± 0.99	17.39^c^ ± 1.24	127.14^c^ ± 1.14	0.15^c^ ± 0.1
C	14.81^a^ ± 1.78	15.62^a^ ± 0.66	38.10^a^ ± 3.1	0.05^a^ ± 0.1

IBW=Initial body weight, FBW=Final body weight, DBWG=Daily body weight gain, FE=Feed efficiency. Notes: The same superscript in the same column indicates that the superscript is not significantly different (p > 0.05)

In addition, the disturbance of pathogenic bacteria can decrease digestibility. In this study, when the temperature reached 37°C, there was a decrease in the consumption of goats that did not receive *dadih*, whereas goats given *dadih* maintained stable consumption levels.

Research conducted by Artanti *et al*. [33] found dry matter consumption of 686.80 ± 24.34 g/head/day in Etawa crossbreed goats (19–22 kg) on a cassava leaf-based diet. A study by Oni *et al*. [34] reported dry matter intake (DMI) of 526, 537, and 528 g/day of dried cassava leaves. The higher DMI observed in this study may be attributed to environmental temperature, ration quality, and the addition of *dadih*. The dry matter consumption exceeded that in studies by Artanti *et al*. [33] and Oni *et al*. [34] and was above the 3.26% body weight recommendation for standard DMI [35], except for the control group. *Dadih*, as a probiotic, supplies beneficial microorganisms to the digestive tract. An increased number of microorganisms improves feed digestion, thereby supporting animal health [8].

The energy content of ruminant feed is determined by total digestible nutrient (TDN); the higher the TDN, the better the feed quality, as more nutrients are absorbed and less excreted. The 4 cc *dadih* treatment significantly increased TDN compared with other treatments (Table 3). The average TDN with *dadih* supplementation ranged from 74% to 77%, well above the 51% required for fattening goats [35].

Regarding cyanide content in cassava leaves, it did not hinder digestibility in this study, as the leaves were harvested at around 3 months of age, wilted mid-day, and fermented overnight before feeding. High cyanide levels reduce palatability. Sun-drying activates linamarase (β-glucosidase), which hydrolyzes cyanogenic glucosides into acetocyanohydrin and glucose. Acetocyanohydrin then breaks down into hydrogen cyanide (HCN) and acetone at pH >5, which evaporate in sunlight, reducing toxicity [36].

The one-night fermentation further reduced cyanide. The process was evident from increased temperature and moisture in the closed containers. LAB developing during fermentation produces β-glucosidase enzymes, especially from *Lactobacillus*, which degrade cyanide [37]. HCN is not problematic when proper drying and fermentation techniques are applied.

### Rumen modulation by probiotics

Probiotics like *dadih* help increase ruminal acidity, inhibiting pathogenic bacteria while suppressing disruptive microorganisms in the digestive system [38]. This supports proper nutrient absorption. Disruptive organisms in the rumen include *E. coli*, *Salmonella*, and Staphylococcus spp. [39].

*Dadih* contains lactic acid-producing bacteria and yeasts, dominated by species such as *Lactobacillus* spp., *Acinetobacter* spp., *Pseudomonas aeruginosa*, *Saccharomyces* spp., and *Bacillus* spp. [11]. Research by Sagaf *et al*. [40] demonstrated that probiotics containing LAB and enzymes enhance daily weight gain, feed consumption, and feed conversion.

Cellulolytic microbes increase cellulose degra-dation rates in cassava leaves. Protease enzymes from proteolytic microbes further break down complex proteins into peptides and amino acids [7]. Lactic acid in *dadih* breaks down glucose into ethanol, lactate, and CO_2_, while yeast accelerates cellulose decomposition.

In this study, feeding started with *dadih*, followed by concentrate. Administered through tube, *dadih* reaches the rumen directly, enriching its microbial ecosystem. The 14% crude protein concentrate likely provided sufficient nutrients to support these micro-bes. The combination of *dadih* and concentrate improved the nutrient supply and rumen function. Consequently, forage intake was optimal in the 4 cc *dadih* group. In contrast, control goats reduced their intake to 37°C.

### DBWG and FE

Climate change presents a major threat to live-stock sustainability. High temperatures lead to behav-ioral and physiological changes, including reduced consumption and weight loss. Identifying adaptive strategies is essential. In this study, 4 cc *dadih* per head per day yielded the highest DBWG (127.14 g/day) and FE (0.15), followed by 8 cc *dadih* (100.00 g/day, FE 0.12). Goats without *dadih* had the lowest DBWG (38.10 g/day) and FE (0.05) (Table 4).

These results indicate that *dadih* supplementation enhances heat stress resilience. Research by Elita [41] reported no significant difference in weight gain bet-ween heat-stressed and non-stressed pea goats (29.14 vs. 31.85 g/day), with DMI at 435.00 and 434.41 g/day and FE at 0.066 and 0.070, respectively. In comparison, FE in this study reached 0.15 with 4 cc *dadih*. According to Mertens and Grant [42] and Khaskheli *et al*. [43], FE depends on palatability, energy level, protein and amino acid concentrations, forage quality, temperature, and metabolic size.

This study suggests that *dadih* can positively influence feed consumption and efficiency under thermal stress. Shabana *et al*. [44] emphasized that feed quality, digestibility, and nutrient adequacy all affect efficiency. In this case, 4 cc *dadih* increased beneficial microbial populations, degraded cellulose and protein, and suppressed harmful bacteria.

### Pathogenic bacteria suppression

*Dadih* as a probiotic contained LAB at 1.2 × 10^10^ CFU/mL. Supplementation helped improve the microbial composition of the gut. Beneficial bacteria in *dadih* included cellulolytic and proteolytic strains that aided fiber and protein digestion, while others controlled pathogens.

Indicators of effective probiotic action included improved digestibility, FE, and weight gain. Probiotics also suppress hindgut pathogens. The 8 cc *dadih* treatment notably reduced *E. coli* in feces (Table 5), confirming its efficacy as a natural antimicrobial.

**Table 5:** *Escherichia coli* with dadih treatment.

Treatment	*Escherichia coli* (10^5^)
A	8 × 10^5A^ ± 3
B	24 × 10^5AB^ ± 3
C	37 × 10^5B^ ± 17

Note: The same superscript in the same column indicates not significantly different (p > 0.05)

Research by Basnet and Kilonzo-Nthenge [45] using Microbact 12E identified *Bacillus sphaericus*, *Yersinia enterocolitica*, and *E. coli* in goat feces. Hidayati *et al*. [46] found that yeast (*Saccharomyces cerevisiae*) reduced *E. coli* in sheep, as acidic conditions inhibited bacterial survival. Kusmiati Kusmiati [47] reported that LAB produces lactic acid, bacteriocins, diacetyl, and hydrogen peroxide – substances that inhibit *Salmonella*, *E. coli*, and *Staphylococcus aureus*.

Isolation of LAB in *dadih* by Pato [48] revealed 36 strains of *Lactobacillus* and *Streptococcus*, while Yuliana *et al*. [49] identified *Lactobacillus* pentosus 124-2. Non-LAB bacteria included *Micrococcus varians*, *Bacillus cereus*, *Staphylococcus saprophyticus*, and the yeast *Endomyces lactis*. According to Kullar *et al*. [11], *Lactobacillus*, *Leuconostoc*, *Lactococcus*, *Streptococcus*, and *Enterococcus* dominate *dadih*.

Roni *et al*. [50] stated that probiotics active in the hindgut include *Bacillus*, *Leuconostoc*, and *Streptococcus*. However, Alugongo *et al*. [51] noted that *L. plantarum* also functions in the hindgut and can alleviate post-weaning disorders. According to Chen *et al*. [52], such disorders involve diarrhea when young goats are exposed to pathogens during feed transition. Wang *et al*. [53] reported that a probiotic mix of *L. acidophilus*, *L. plantarum*, and *Enterococcus faecium* (3.0 × 10^9^ CFU/kg) mitigated aflatoxin effects and improved sheep growth.

This study demonstrated that *dadih* bacteria adhere to both foregut (rumen, reticulum, omasum) and hindgut, enhancing FE and reducing the pathogenic load (Table 5).

### Hematological parameters following *dadih* treatment

Hematological values remained within normal ranges across all treatments, indicating that *dadih* supplementation was safe (Table 6). Research by Raja *et al*. [54] using 1% *Bacillus pumilus* in Etawa goats reported significant effects on hemoglobin (Hb), red blood cell (RBC), white blood cell (WBC), and hema-tocrit (HCT) (p < 0.05), with values of 8.13–9.50 g/dL (Hb), 8.3–9.2 × 10^6^/μL (RBC), 10.75–13.30 × 10^3^/μL (WBC), and 25.00%–34.00% (HCT).

**Table 6:** Hematological parameters of Sapera goats after *dadih* treatment.

Treatment	MCH	MCHC	MCV	Hb	RBC	WBC	HCT
A	6.933^a^ ± 0.322	39.9^a^ ± 0	17.567^a^ ± 1.001	8.467^a^ ± 0.551	12.226^a^ ± 1.234	12.033^a^ ± 0.551	21.433^a^ ± 1.250
B	7.233^a^ ± 1.021	41.667^a^ ± 3.669	17.4^a^ ± 0.819	8.033^a^ ± 0.635	11.146^a^ ± 1.500	12.4^a^ ± 0.8	19.333^a^ ± 1.792
C	6.733^a^ ± 0.404	39.367^a^ ± 0.924	17.167^a^ ± 0.862	9.133^a^ ± 0.702	14.22^a^ ± 1.221	11.933^a^ ± 0.764	23.233^a^ ± 1.206

MCH=Mean corpuscular hemoglobin, MCHC=Mean corpuscular hemoglobin concentration, MCV=Mean corpuscular volume, Hb=Hemoglobin, RBC=Red blood cell, WBC=White blood cell, HCT=Hematocrit. Note: The same superscript in the same column indicates not significantly different (p > 0.05)

Yahya *et al*. [55] found that up to 30% sugarcane bagasse supplemented with probiotics had no adverse effect on Red Sokoto goats. Their hematological values were Hb 10.33 g/dL, RBC 8.5 × 10^6^/μL, WBC 9.33 × 10^3^/μL, mean corpuscular volume 26.33 fL, mean corpuscular hemoglobin 12.04 pg, and mean corpuscular hemoglobin concentration (MCHC) 46.12 pg.

The Hb values in this study fall within the 7–15 g/dL range reported by Daramola *et al*. [56], reflecting nor-mal nutrient metabolism and oxygen-carrying capacity. RBC counts (within 8–18 × 10^6^/μL) indicate the absence of anemia [57–59]. WBC counts were also within normal limits (10.45–15.91 × 10^3^/μL) [59], signifying a healthy immune response. Elevated WBCs are common in young ruminants in tropical environments due to higher parasitic loads.

HCT values (24%–48%) [60] confirm that the animals were healthy. MCHC values in this study (8.67–10.67 × 10^3^/μL) also fall within the reported normal range of 6.8–20.1 × 10^3^/μL [56].

## CONCLUSION

This study demonstrated the potential of *dadih*, a traditional Indonesian fermented buffalo milk, as an effective probiotic supplement for enhancing the health and productivity of heat-stressed Sapera goats. The dominant bacterial strain isolated from *dadih* was identified as *L. plantarum* JCM 1149, which exhibited *in vitro* antibacterial activity against *E. coli* and *Salmonella*, with inhibition zones of 9.3 mm and 9.5 mm, respectively.

Among the treatments, supplementation with 4 cc of *dadih* per day resulted in the highest ADG (127.14 g/day), best FE (0.15), and improved DMI and nutrient digestibility. These enhancements were attributed to the probiotic’s ability to stabilize rumen microbiota, enhance cellulose and protein degra-dation, and suppress pathogenic bacteria. Notably, fecal *E. coli* populations were significantly reduced in the 8 cc treatment group, indicating dose-dependent antimicrobial effects. Hematological parameters remained within physiological norms across all groups, confirming the safety of *dadih* supplementation.

The strengths of this study lie in its novel inte-gration of ethnoveterinary knowledge with controlled experimental validation, its multi-parameter assessment (microbial, performance, hematological), and the use of *dadih* as a culturally relevant, cost-effective probiotic.

However, limitations include the small sample size (n = 5 per group), the short duration of the feeding trial (4 weeks), and the lack of longitudinal monitoring to evaluate long-term effects on health and productivity. In addition, while this study confirmed microbial activity and performance outcomes, it did not investigate host gene expression, rumen microbiome composition, or systemic immune modulation.

Future studies should explore the molecular mechanisms underlying the probiotic-host interaction, including metagenomic analysis of gut microbiota and the identification of specific metabolic pathways influenced by *dadih*. Longer-term trials with larger populations, under varying environmental stress conditions, would further validate these findings. Invest-igating the efficacy of *dadih* in other livestock species and developing standardized probiotic formulations could support its broader application in climate-resilient animal agriculture.

## AUTHORS’ CONTRIBUTIONS

NG: Conceptualized, methodology, investigation, analyzed and interpreted data, and drafted, reviewed, and edited the manuscript. NBK and EM: Methodology, investigation, analyzed and interpreted data, and reviewed and edited the manuscript. YYL: Methodology, formal analysis, and reviewed and edited the manuscript. All authors have read and approved the final manuscript.

## References

[1] Pramono A, Altiara D.N.P, Cahyadi M (2023). The effect of differences in lactation period and milking time on milk production and quality of Saanen Etawa Crossbreed Goats (Sapera). IOP Conf. Ser. Earth Environ. Sci.

[2] Suranindyah Y, Widyobroto B.P, Astuti S.D, Murti T.W, Adiarto A (2020). Lactation characteristic of etawah crossed breed goats under intensive management. Bul. Peternakan.

[3] Yudhanto F, Rachmawati P, Agustin C (2023). Effect of feeding size of cassava leaf by chopper machine on milk production of goat at Kemirikebo village. E3S Web. Conf.

[4] Sejian V, Silpa M.V, Reshma Nair M.R, Devaraj C, Krishnan G, Bagath M, Chauhan S, Suganthi R, Fonseca V, Konig S, Gaughan J, Dunshea F, Bhatta R (2021). Heat stress and goat welfare:Adaptation and production considerations. Animals (*Basel*).

[5] Farias Machado N.A, Filho J.A.D.B, de Oliveira K. P.L, Parente M.D.O.M, de Siqueira J.C, Pereira A.M, Santos A.R.D, Sousa J.M.S, Rocha K.S, Viveiros K.K, Costa C.D.S (2020). Biological rhythm of goats and sheep in response to heat stress. Biol. Rhythm Res.

[6] Cai L, Hartanto R, Zhang J, Qi D (2021). *Clostridium butyricum* improves rumen fermentation and growth performance of heat-stressed goats *in vitro* and *in vivo*. Animals (*Basel*).

[7] Arnold M, Rajagukguk Y.V, Gramza-Michałowska A (2021). Characterization of *dadih*:Traditional fermented buffalo milk of Minangkabau. Beverages.

[8] Ginting N (2018). Comparison of isolate *dadih* with yeast *dadih* in improving nutrition quality of Cassava Waste (CW). IOP Conf. Ser. Earth Environ. Sci.

[9] Abid S, Farid A, Abid R, Rehman M.U, Alsanie W.F, Alhomrani M, Alamri A.S, Asdaq S.M.B, Hefft D.I, Saqib S, Muzammal M, Morshedy S.A, Alruways M.W, Ghazanfar S (2022). Identification, biochemical characterization, and safety attributes of locally isolated *Lactobacillus fermentum* from *Bubalus bubalis* (buffalo) milk as a probiotic. Microorganisms.

[10] Wirawati C.U, Sudarwanto M.B, Lukman D.W, Wientarsih I (2017). Characteristic and development of cow's milk *dadih* as an alternate of buffalo's milk *dadih*. Wartazoa.

[11] Kullar R, Goldstein E.J, Johnson S, McFarland L.V (2023). *Lactobacillus bacteremia* and probiotics:A review. Microorganisms.

[12] Iswarin R, Fani F, Pambudi A.W (2016). Particle size's effect of application forage processing technology on consumption efficiency, palatability and digestibility of local goat. Anim. Prod.

[13] Jiwuba P.C, Jiwuba L.C (2020). Productive and physiological response of small ruminants fed cassava (*Manihot esculenta* Crantz) and cassava by-products in their diets:A review. Bulg. J. Anim. Husb.

[14] Jiwuba P.C, Jiwuba L.C, Ogbuewu I.P, Mbajiorgu C.A (2021). Enhancement values of cassava by-product diets on production and haemato-biochemical indices of sheep and goats:A review. Trop. Anim. Health Prod.

[15] Ihtifazhuddini F.M.T, Batan I.W, Nindhia T.S (2021). Feeding local forage supplemented with indigofera and probiotics on the erythrocyte profile of Boerka goats [Pemberian pakan hijauan lokal yang disuplementasi indigofera dan probiotik terhadap profil eritrosit kambing boerka]. Indones. Med. Vet.

[16] Ary E, Dadrasnia A, Ameen F, Alwakeel S, Ismail S (2021). Antimicrobial screening of lactic acid bacteria isolated from fermented milk buffalo (*Dadih*). Int. J. Sci. Res. Publ.

[17] Yu J, Wang W.H, Menghe B.L.G, Jiri M.T, Wang H.M, Liu W.J, Bao Q.H, Lu Q, Zhang J.C, Wang F, Xu H.Y, Sun T.S, Zhang H.P (2011). Diversity of lactic acid bacteria associated with traditional fermented dairy products in Mongolia. J. Dairy Sci.

[18] Gao M.L, Hou H.M, Teng X.X, Zhu Y.L, Hao H.S, Zhang G.L (2017). Microbial diversity in raw milk and traditional fermented dairy products (Hurood cheese and Jueke) from Inner Mongolia, China. Genet. Mol. Res.

[19] Zhang W.Y, Yun Y.Y, Sun T.S, Menghe B, Zhang H.P (2008). Isolation and identification of dominant microorganisms involved in naturally fermented goat milk in Haixi region of Qinghai, China. Ann. Microbiol.

[20] Jans C, Bugnard J, Njage P.M.K, Lacroix C, Meile L (2012). Lactic acid bacteria diversity of African raw and fermented camel milk products reveals a highly competitive, potentially health-threatening predominant microflora. LWT Food Sci. *Technol*.

[21] Mechai A, Debabza M, Kirane D (2014). Screening of technological and probiotic properties of lactic acid bacteria isolated from Algerian traditional fermented milk products. Int. Food Res. J.

[22] Abed T.A (2013). Evaluation of methods for the extraction and purification of DNA of cultured *Lactobacillus* colony isolated from dairy products. Int. J. Appl. Microbiol.

[23] Yu J, Wang H.M, Zha M.S, Qing Y.T, Bai N, Ren Y, Xi X.X, Liu W.J, Menghe L.G, Zhang H.P (2015). Molecular identification and quantification of lactic acid bacteria in traditional fermented dairy foods of Russia. J. Dairy Sci.

[24] Yu J, Sun Z, Liu W, Zhang J, Sun T, Bao Q, Zhang H (2009). Rapid identification of lactic acid bacteria isolated from home-made fermented milk in Tibet. J. Gen. Appl. Microbiol.

[25] Liu W, Xi X, Sudu Q, Kwok L, Guo Z, Huo Q, Menhe B, Sun T, Zhang H (2015). High-throughput sequencing reveals microbial community diversity of Tibetan naturally fermented yak milk. Ann. Microbiol.

[26] Ren L, Suo H (2017). Molecular identification of lactic acid bacteria isolated from the traditional fermented yak yogurt in Western Sichuan region. Adv. Comput. Sci. Res.

[27] Shangpliang H.N.J, Rai R, Keisam S, Jeyaram K, Tamang J.P (2018). Bacterial community in naturally fermented milk products of Arunachal Pradesh and Sikkim of India analyzed by high-throughput amplicon sequencing. Sci. Rep.

[28] Ehsani A, Hashemi M, Afshari A, Aminzare M (2018). Probiotic white cheese production using coculture with *Lactobacillus* species isolated from traditional cheeses. Vet. World.

[29] Zendeboodi F, Khorshidian N, Mortazavian A.M, da Cruz A.G (2020). Probiotic:Conceptualization from a new approach. Curr. Opin. Food Sci.

[30] Salvetti E, Harris H.M, Felis G.E, O'Toole P.W (2018). Comparative genomics of the genus *Lactobacillus* reveals robust phylogroups that provide the basis for reclassification. Appl. Environ. Microbl.

[31] Girma A, Aemiro A (2021). Antibacterial activity of lactic acid bacteria isolated from fermented Ethiopian traditional dairy products against food spoilage and pathogenic bacterial strains. J. Food Qual.

[32] Shafique B, Ranjha M.M.A.N, Murtaza M.A, Walayat N, Nawaz A, Khalid W, Mahmood S, Nadeem M, Manzoor M.F, Amwwe K, Aadil R.M, Ibrahim S.A (2022). Recent trends and applications of nanoencapsulated bacteriocins against microbes in food quality and safety. Microorganisms.

[33] Artanti O.W, Ridla M, Khotijah L (2019). The use of cassava leaves *(Manihot esculenta*) with different processing on the performance of male peranakan etawa goats [Penggunaan daun ubi kayu (*Manihot esculenta*) dengan pengolahan berbeda terhadap performa kambing peranakan etawa Jantan]. J. Ilmiah Peternakan Terpadu.

[34] Oni A.O, Arigbede O.M, Oni O.O, Onwuka C.F. I, Anele U.Y, Oduguwa B.O, Yusuf K.O (2010). Effects of feeding different levels of dried cassava leaves (*Manihot esculenta*, Crantz) based concentrates with Panicum maximum basal on the performance of growing West African Dwarf goats. Livestock Sci.

[35] Abdalhamed A.M, Ghazy A.A, Zeedan G.S.G (2021). Studies on multidrug-resistance bacteria in ruminants with special interest on antimicrobial resistances genes. Adv. Anim. Vet. Sci.

[36] Githunguri C.M, Gatheru M, Ragwa S.M (2017). Status of cassava processing and challenges in the coastal, eastern and western regions of Kenya. Handbook on Cassava. Nova Science Publisher, Inc. New York.

[37] Fawole A.O (2019). Selection of Lactic Acid Bacteria for Use as Starter Cultures in Lafun Production and their Impact on Product Quality and Safety (Doctoral dissertation, University of Reading).

[38] Kuliahsari D.E, Sari I.N.I, Estiasih T (2021). Cyanide detoxification methods in food:A review. IOP Conf. Ser. Earth Environ. Sci.

[39] Jacob O.A, Anuoluwa O.E, Raimi M.O (2021). Potential toxic levels of cyanide and heavy metals in cassava flour sold in selected markets in Oke Ogun community, Oyo State, Nigeria. Front. Sustain. Food Syst.

[40] Sagaf S, Latasiki M.A, Padang P (2023). Comparison of Body Weight Gain of Female Bean Goats Exposed and Unexposed to the Sun [Perbandingan pertambahan bobot badan kambing kacang betina yang terpapar dan tidak terpapar matahari]. Agroland J. Ilmu-ilmu Pertanian.

[41] Elita A.S (2006). Comparative Study of General Appearance and Feed Digestibility in Local Goats and Sheep [Studi Perbandingan Penampilan Umum Dan Kecernaan Pakan Pada Kambing Dan Domba Lokal]. Agris. FAO, Paris.

[42] Mertens D.R, Grant R.J (2020). Digestibility and intake. Forages.

[43] Khaskheli A.A, Khaskheli M.I, Khaskheli A.J, Khaskheli A.A (2020). Significance of feeding practices for small ruminants:A reveiw. Agric. Rev.

[44] Shabana I.I, Albakri N.N, Bouqellah N.A (2021). Metagenomic investigation of faecal microbiota in sheep and goats of the same ages. J. Taibah Univ. Sci.

[45] Basnet A, Kilonzo-Nthenge A (2024). Antibiogram profiles of pathogenic and commensal bacteria in goat and sheep feces on smallholder farm. Front. Antibiot.

[46] Hidayati Y.A, Benito A.K.T, Harlia E (2013). Analysis of bacterial count and identification of bacteria in liquid fertilizer from sheep feces with the addition of *Saccharomyces cerevisiae* [Analisis jumlah bakteri dan identifikasi bakteri pada pupuk cair dari feses domba dengan penambahan *Saccharomyces cerevisiae*]. J. Ilmu Ternak.

[47] Kusmiati Kusmiati M.A (2002). Bacteriocin activity of *Leuconostoc mesenteroides* Pbac1 bacteria in various media [Aktivitas bakteriosin dari bakteri leuconostoc mesenteroides Pbac1 pada berbagai media]. J. Makara Kesehatan.

[48] Pato U (2003). Potential of lactic acid bacteria isolated from curd to lower the risk of cancer [Potensi bakteri asam laktat yang diisolasi dari *dadih* untuk menurunkan resiko penyakit kanker]. J. Nat. Indones.

[49] Yuliana T, Tyano F.N, Harlina P.W, Cahyana Y, Marta H, Krama A (2023). Characterizing probiotic lactic acid bacteria from buffalo milk fermentation (*Dadih*) for beef Biopreservation. Appl. Sci.

[50] Roni P, Jamarun N, Sucitra L.S (2023). Probiotics for ruminasia [Probiotik untuk ruminansia]. CV. Adanu Abimata, Indramayu, Jawa Barat, Indonesia.

[51] Alugongo G.M, Xiao J.X, Chung Y.H, Dong S.Z, Li S.L, Yoon I, Wu Z.H, Cao Z.J (2017). Effects of *Saccharomyces cerevisiae* fermentation products on dairy calves:Performance and health. J. Dairy Sci.

[52] Chen K, Liu Y, Cheng Y, Yan Q, Zhou C, He Z, Zeng J, He J, Tan Z (2020). Supplementation of *Lactobacillus plantarum* or *Macleaya cordata* extract alleviates oxidative damage induced by weaning in the lower gut of young goats. Animals (*Basel*).

[53] Wang J, Lin L, Jiang Q, Huang W, Liu N (2019). Effect of supplemental lactic acid bacteria on growth performance, glutathione turnover and aflatoxin B1 removal in lambs. Czech J. Anim. Sci.

[54] Raja D.N.L, Raguati R, Insulistyowati A (2022). Effect of using rubber leaves as a forage source supplemented with probiotics on the blood hemogram profile of peranakan etawah goats [Pengaruh penggunaan daun karet sebagai sumber hijauan yang disupplementasi probiotik terhadap profil hemogram darah kambing peranakan etawah]. J. Ilmiah Ilmu-Ilmu Peternakan.

[55] Yahya M.M, Umar F, Yakubu A.K (2020). Effect of feeding graded levels of probiotic supplemented sugarcane bagasse on performance and haematological parameters of red sokoto goats. J. Agric. Environ.

[56] Daramola J.O, Adeloye A.A, Fatoba T.A, Soladoye A.O (2005). Haematological and biochemical parameters of West African Dwarf goats. Livestock Res. Rural Dev.

[57] Weiss D.J, Wadrobe K.J (2010). Schlam's Veterinary Hematology.

[58] Waziri M.A, Ribadu A.Y, Sivachelvan N (2010). Changes in the serum proteins, hematological and some serum biochemical profiles in the gestation period in the Sahel goats. Vet. arhiv.

[59] Dunn J.K (2000). Textbook of Small Animal Medicine. WB Saunders, New York.

[60] Gregg L.V.D (2000). Hematology Techniques and Concepts for Veterinary Technicians. John Wiley &Sons Ltd., The Atrium, Southern Gate, Chichester, West Sussex, PO19 OX42DQ, UK.

